# Taming Microglia in Alzheimer’s Disease: Exploring Potential Implications of Choline Alphoscerate via α7 nAChR Modulation

**DOI:** 10.3390/cells13040309

**Published:** 2024-02-07

**Authors:** Anna Flavia Cantone, Chiara Burgaletto, Giulia Di Benedetto, Anna Pannaccione, Agnese Secondo, Carlo Maria Bellanca, Egle Augello, Antonio Munafò, Paola Tarro, Renato Bernardini, Giuseppina Cantarella

**Affiliations:** 1 Section of Pharmacology, Department of Biomedical and Biotechnological Sciences, University of Catania, 95123 Catania, Italy; anna.cantone@phd.unict.it (A.F.C.); chiaraburg@hotmail.it (C.B.); uni318437@studium.unict.it (C.M.B.); uni365053@studium.unict.it (E.A.); antonio.munafo.91@gmail.com (A.M.); paolatarro@hotmail.com (P.T.); bernardi@unict.it (R.B.); gcantare@unict.it (G.C.); 2 Clinical Toxicology Unit, University Hospital of Catania, 95123 Catania, Italy; 3 Division of Pharmacology, Department of Neuroscience, Reproductive and Odontostomatological Sciences, University of Naples Federico II, 80131 Naples, Italy; pannacio@unina.it (A.P.); agnese.secondo@unina.it (A.S.)

**Keywords:** acetylcholine, cholinergic transmission, microglia, inflammatory response

## Abstract

Alzheimer’s disease (AD), marked by cognitive impairment, predominantly affects the brain regions regulated by cholinergic innervation, such as the cerebral cortex and hippocampus. Cholinergic dysfunction, a key contributor to age-related cognitive decline, has spurred investigations into potential therapeutic interventions. We have previously shown that choline alphoscerate (α-GPC), a cholinergic neurotransmission-enhancing agent, protects from Aβ-mediated neurotoxicity. Herein, we investigated the effects of α-GPC on the microglial phenotype in response to Aβ via modulation of the nicotinic alpha-7 acetylcholine receptor (α7 nAChR). BV2 microglial cells were pre-treated for 1 h with α-GPC and were treated for 24, 48, and 72 h with Aβ_1–42_ and/or α-BTX, a selective α7nAchR antagonist. Fluorescent immunocytochemistry and Western blot analysis showed that α-GPC was able to antagonize Aβ-induced inflammatory effects. Of note, α-GPC exerted its anti-inflammatory effect by directly activating the α7nAChR receptor, as suggested by the induction of an increase in [Ca^2+^]_i_ and Ach-like currents. Considering that cholinergic transmission appears crucial in regulating the inflammatory profiles of glial cells, its modulation emerges as a potential pharmaco-therapeutic target to improve outcomes in inflammatory neurodegenerative disorders, such as AD.

## 1. Introduction

Alzheimer’s disease (AD) represents the most common age-related neurodegenerative disorder associated with memory deficits and cognitive decline [1]. Critical brain regions for the maintenance of cognitive processes include the cerebral cortex and hippocampus, whose cholinergic innervations are mainly provided by the basal forebrain cholinergic nuclei (BFCN) [2,3]. The dysfunction of cholinergic neurotransmission driven by the selective degeneration of BFCN has led to the formulation of the “cholinergic hypothesis of age-related cognitive dysfunction” [4]. Recognition of the pivotal role of altered cholinergic transmission in disease pathophysiology has spurred the development of therapeutic strategies aimed at restoring cholinergic function. 

Cholinergic precursors represent the first potential approach aimed to counteract the cholinergic dysfunction and cognitive decline that occur in various forms of dementia [5]. Among them, choline alphoscerate (L-alpha-glycerylphosphoxycholine; α-GPC), given its high choline content (41% choline by weight) and ability to cross the blood–brain barrier, is one of the most used sources of choline [6]. Indeed, it has been found to be effective in improving the synthesis and release of acetylcholine [5]. 

The activation of nicotinic acetylcholine receptors (nAChRs) by acetylcholine in the frontal cortex of the brain has been shown to be essential for functions such as attention and working memory [7,8]. Specifically, the nicotinic alpha-7 acetylcholine receptor (α7 nAChR), a ligand-activated ion channel, has been demonstrated to play an important role in AD [9]. Indeed, in addition to regulating neuronal plasticity and turnover, α7 nAChR also has vital functions in microglia [10].

Microglial cells are the primary effectors of the innate immune system within the brain [11,12]. Upon activation, microglia can display a broad spectrum of phenotypes, ranging from the classical pro-inflammatory phenotype M1 to the alternative anti-inflammatory phenotype M2 [13], with α7 nAChR playing a crucial role in mediating the above-mentioned contexts [14]. 

Considering the potential beneficial role of cholinergic precursors at the crossroads between neurodegeneration and neuroinflammation, in the present study, we attempted to investigate the mechanism underlying such effects using an in vitro model of AD.

Specifically, we investigated the effects of α-GPC, used as an indirect agonist of α7 nAchR, on Aβ-induced microglial phenotypic switching in the BV2 murine microglial cell line. In addition, we used a selective α7 nAchR antagonist, α-bungarotoxin, to substantiate the receptor’s direct involvement in mediating this mechanism. 

## 2. Materials and Methods

### 2.1. Drugs and Chemicals

All media were obtained from Thermo Fisher Scientific, Inc., Waltham, MA, USA. L-alpha-glycerylphosphoxycholine (α-GPC) was provided by Italfarmaco, Milano, Italy. Lyophilised amyloid-β protein fragment 1–42 was obtained from Sigma-Aldrich, St. Louis, MO, USA. All other chemicals were of the highest commercial grade.

### 2.2. Preparation of Aβ_1–42_ Oligomers

Aβ_1–42_ oligomers were prepared as previously described [15]. Briefly, lyophilized Aβ_1–42_ peptide was first dissolved in 1,1,1,3,3,3-hexafluoro-2-propanol (HFIP; Sigma-Aldrich) under a fume hood to a final concentration of 1 mM. The solution was aliquoted and incubated at room temperature (RT) for 2 h to allow monomerization. A SpeedVac centrifuge (800× *g*, RT) was used to remove traces of HFIP, and the clear peptide film was stored over desiccant at −80 °C. For oligomerization, the aliquoted peptide film was dissolved in dimethyl sulfoxide (DMSO) to 5 mM. To obtain the oligomeric form of Aβ_1–42_, the peptide in DMSO was diluted directly into sterile phosphate-buffered saline (PBS, 1×) at 100 μM and incubated at 4 °C for 12 h. After incubation, the Aβ_1–42_ samples were either used immediately for treating cells or were aliquoted and stored at –20 °C until use.

### 2.3. Cell Cultures 

The BV2 murine microglial cell line was obtained from AcceGen (AcceGen Biotech, Fairfield, NJ, USA; Cat. ABC-TC212S; Accession Number: CVCL_0182). The cells were cultured in Dulbecco’s Modified Eagle Medium (DMEM; High Glucose) supplemented with 10% (*v*/*v*) fetal bovine serum, 100 g/mL penicillin, 100 g/mL streptomycin, 1% (*v*/*v*) sodium pyruvate, and 2 mM glutamine. The cells were maintained in an atmosphere of 5% CO_2_ and 95% humidified air at 37 °C.

### 2.4. Cell Viability Test

Cell viability was determined using a 3-[4,5 dimethylthiazol-2-yl]-2,5-diphenyltetrazolium bromide (MTT) assay. A total of 5 × 10^3^ cells per well were plated on 96-well plates. The culture medium was changed to a medium containing MTT (Sigma-Aldrich, Milan, Italy), and cell viability was quantified by measuring the reduction of the MTT solution (0.5 mg/mL). Following incubation for 3 h at 37 °C, the solution was removed, and dimethyl sulfoxide (DMSO) was added for cell lysis and solubilization of the blue formazan crystals resulting from MTT reduction by the mitochondrial activity of viable cells. A VarioskanTM Flash Multimode Reader was used to measure the optical density of the supernatants at 545 nm. The data were expressed as the mean percentage of viable cells compared to the control. The experiments were performed in triplicate at least twice.

### 2.5. Western Blot Analysis 

For protein extraction and Western blot analysis, the cells were lysed in buffer containing 150 mM NaCl, 5 mM EDTA, 50 mM Tris-HCl (pH 7.5), 1 mM Na_3_VO_4_, 1 mM acid phenyl-methyl-sulphonyl-fluoride, 30 mM sodium pyrophosphate, 50 mM NaF, 5 µg/mL aprotinin, 2 µg/mL leupeptin, 1 µg/mL pepstatin, 10% glycerol, and 0.2% TritonTM X-100. The homogenates were then centrifuged at 14,000 rpm for 10 min at 4 °C. The protein concentration of the supernatant was determined using the Bradford method [16]. Equal amounts of protein (30 µg) were resolved on 8–12% SDS-PAGE gels and transferred onto Hybond ECL nitrocellulose membranes (GE Healthcare, Little Chalfont, UK). The membranes were blocked for 1 h at RT with 5% non-fat dry milk plus 0.05% Tween 20. For the primary antibody reactions, a rabbit anti-IL-10 antibody (Abbiotec, San Diego, CA, USA, 250713; 1:250), a rabbit anti-TNF-α antibody (Novus Biologicals, Littleton, CO, USA, NB600-587; 1:1000), or rabbit anti-α7 nAchR (Abcam, Cambridge, UK, ab216485; 1:250) were added to the membranes and incubated overnight at 4 °C on an orbital shaker. Then, the membranes were washed with PBS plus 0.05% Tween 20 (PBS-T) and were probed with the appropriate horseradish peroxidase-conjugated secondary antibody (Amersham Life Science, Buckinghamshire, UK) for 1 h at RT. β-actin (Santa Cruz Biotechnology Inc., Santa Cruz, CA, USA, sc-47778; 1:500) was used as a control to validate the amount of protein loaded in the gels. After washing three times with PBS-T, detection was performed using an ECL chemiluminescence assay (Amersham Life Science). The protein bands were scanned with the iBright FL1500 Imaging System (Thermo Fisher Scientific). The densitometric analysis of band intensity was conducted on immunoblots using IMAGE J software version 1.53v (https://imagej.nih.gov/ij/, accessed on 9 Octber 2023).

### 2.6. Fluorescent Immunocytochemistry 

After treatment, the BV2 cells were fixed for 15 min in 4% paraformaldehyde, permeabilized for 7 min with 0.1% Triton X-100, and then blocked for 30 min with 1% BSA. The cells were incubated for 1 h at RT with mouse anti-CD86 (Santa Cruz Biotechnology Inc., Santa Cruz, CA, USA, sc-28347, 1:250), mouse anti-CD68 (Santa Cruz Biotechnology Inc., sc-20060; 1:250), a rabbit anti-IL-10 antibody (Abbiotec, 250713; 1:200), a rabbit anti-TNF-α antibody (Novus Biologicals, NB600-587; 1:100), or a rabbit anti-α7 nAchR (Abcam, ab216485; 1:500). After washing in PBS three times for 5 min each, the cells were incubated for 1 h at RT in the dark with the appropriate fluorescent-labelled secondary antibodies (Alexa Fluor 488 donkey anti-mouse (Thermo Fisher Scientific), Alexa Fluor 546 donkey anti-rabbit (Thermo Fisher Scientific), or Alexa Fluor 488 donkey anti-rabbit (Thermo Fisher Scientific)). Finally, for nuclear staining and the stabilization of fluorescent signals, the slides were covered in mounting medium (Fluoroshield with DAPI; Sigma-Aldrich, Milan, Italy) and secured with a coverslip. Fluorescence images were captured using a Zeiss Observer.Z1 microscope equipped with the Apotome.2 acquisition system (Zeiss LSM 700, Jena, Germany).

### 2.7. [Ca^2+^]_i_ Measurements

[Ca^2+^]_i_ was measured using single-cell Fura-2 acetoxymethyl-ester (AM) video imaging, as previously described [17]. The BV2 cells, placed on glass coverslips, were loaded with 10 μmol/L Fura-2AM for 30 min at 37 °C in normal Krebs solution containing 5.5 mM KCl, 160 mM NaCl, 1.2 mM MgCl_2_, 1.5 mM CaCl_2_, 10 mM glucose, and 10 mM HEPES-NaOH (pH 7.4). [Ca^2+^]_i_ was measured using a live-imaging system composed of an inverted Zeiss Axiovert 200 microscope (Carl Zeiss, Goettingen, Germany), a MicroMax 512BFT cooled CCD camera (Princeton Instruments, Trenton, NJ, USA), a LAMBDA10-2 filter wheeler (Sutter Instruments, Novato, CA, USA), and Meta-Morph/MetaFluor Imaging System software version 7.8 (Universal Imaging, West Chester, PA, USA). After loading, the samples were alternatively illuminated at 340 nm and 380 nm wavelengths. The drug effect on [Ca^2+^]_i_ was evaluated as Δ% peak increase over basal values in the absence or presence of α-bungarotoxin. The BV2 cells were treated with the toxin for 5 min before the registration and were then analyzed.

### 2.8. Patch-Clamp Electrophysiology 

The α7 nAChR currents were recorded from the BV2 cells using the patch-clamp technique in a whole-cell configuration using a commercially available amplifier (Axopatch200B (Molecular Devices, San Jose, CA, USA)), and the data were acquired using a Digidata1322A acquisition system (Molecular Devices) and pCLAMP software, version 10.5 (Molecular Devices, Burlingame, CA, USA). The peak current amplitude and charge movement (area under curve, AUC) induced by agonist application were measured using ClampFit 10 (Molecular Devices). The borosilicate microelectrode (resistance of 4–5 MΩ) was prepared with a puller (Narishige, PC-10, Tokyo, Japan). The dialyzing pipette solution contained the following (in mM): 100 Cs-gluconate, 10 TEA, 20 NaCl, 1 Mg-ATP, 0.1 CaCl_2_, 2 MgCl_2_, 0.75 EGTA, and 10 HEPES, adjusted to a pH of 7.2 using CsOH. The cells were perfused with external Ringer’s solution containing the following (in mM): 126 NaCl, 1.2 NaHPO_4_, 2.4 KCl, 2.4 CaCl_2_, 1.2 MgCl_2_, 10 glucose, and 18 NaHCO_3_, with a pH of 7.4. The holding potential was maintained at −70 mV to record the Ach currents. The currents were filtered at 2 kHz and digitized at 10 kHz. The drugs were applied using a hand-held pipette at the following concentrations: 1 mM acetylcholine, 1 mM α-GPC, and 10 nM α-bungarotoxin.

### 2.9. Statistical Evaluation

All the experiments were run in triplicate. The data were analyzed using the one-way ANOVA test, followed by the Bonferroni post-hoc test. Statistical evaluation was performed using standard computer software (SPSS software package, ver. 23.0, SPSS Inc., Chicago, IL, USA). Statistical significance was set at a *p* < 0.05. The graph design and statistical analyses were performed using Graph Pad Prism (Ver. 8, La Jolla, CA, USA).

## 3. Results

### 3.1. Alpha-GPC Counteracts Aβ-Induced Toxicity in BV2 Microglial Cells

To assess α-GPC’s potential protective role on BV2 microglial cells against Aβ toxicity, viability experiments were conducted using the MTT assay. Initially, we tested the concentration-related effect (ranging from 1 pM to 100 μM) of α-GPC alone on BV2 microglial cell viability following treatment for 24, 48, and 72 h. α-GPC did not significantly affect cell proliferation, nor did it induce cell toxicity at low concentrations. Nevertheless, at concentrations of 25, 50, and 100 μM, α-GPC showed toxic effects, as demonstrated by a significant decrease in cell viability at all the time points studied.

**Figure 1:**
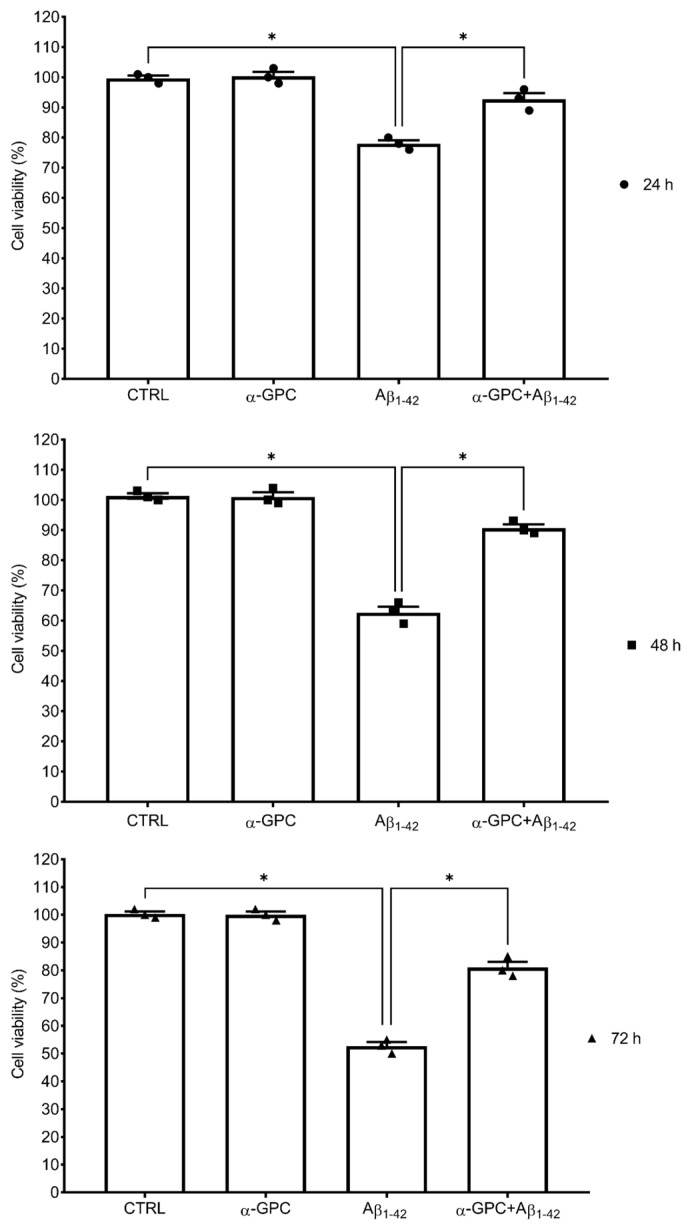
α-GPC mitigates the Aβ_1–42_-induced detrimental effect on BV2 microglial cells. The cell viability (%) of BV2 microglial cells pre-treated for 1 h with α-GPC (1 μM) and treated for 24, 48, or 72 h with Aβ_1–42_ (5 μM). The vertical bars represent the means ± S.E.M. One-way ANOVA and the Bonferroni post-hoc test were used for statistical analysis. * *p* < 0.05.

Consistent with these observations, α-GPC at 1 µM was chosen for the subsequent experiments. To explore whether α-GPC exerts a protective effect against Aβ-related toxicity on BV2 cells, the cells were pre-treated for 1 h with α-GPC 1 µM and were treated for 24, 48, and 72 h with α-GPC 1µM and/or Aβ_1–42_ (5 µM). α-GPC was able to mitigate the Aβ_1–42_-induced detrimental effect on BV2 microglial cells at all the time points studied (Figure 1). 

### 3.2. α-GPC Blunts the Aβ-Induced Inflammatory Phenotype in BV2 Microglial Cells

To investigate whether the protective effect of α-GPC on BV2 microglia exposed to Aβ was also associated with changes in microglia phenotype, the expression of phenotype-associated molecules was analyzed by means of immunofluorescence analysis. The BV2 cells were treated with Aβ_1–42_ (5 µM) for 48 h, either alone or in combination with α-GPC 1 µM (1 h of pre-treatment and treatment for 48 h). The time point of 48 h was selected for the subsequent experiments, as it represents the time point at which the microglial inflammatory phenotypes were activated [18].

**Figure 2:**
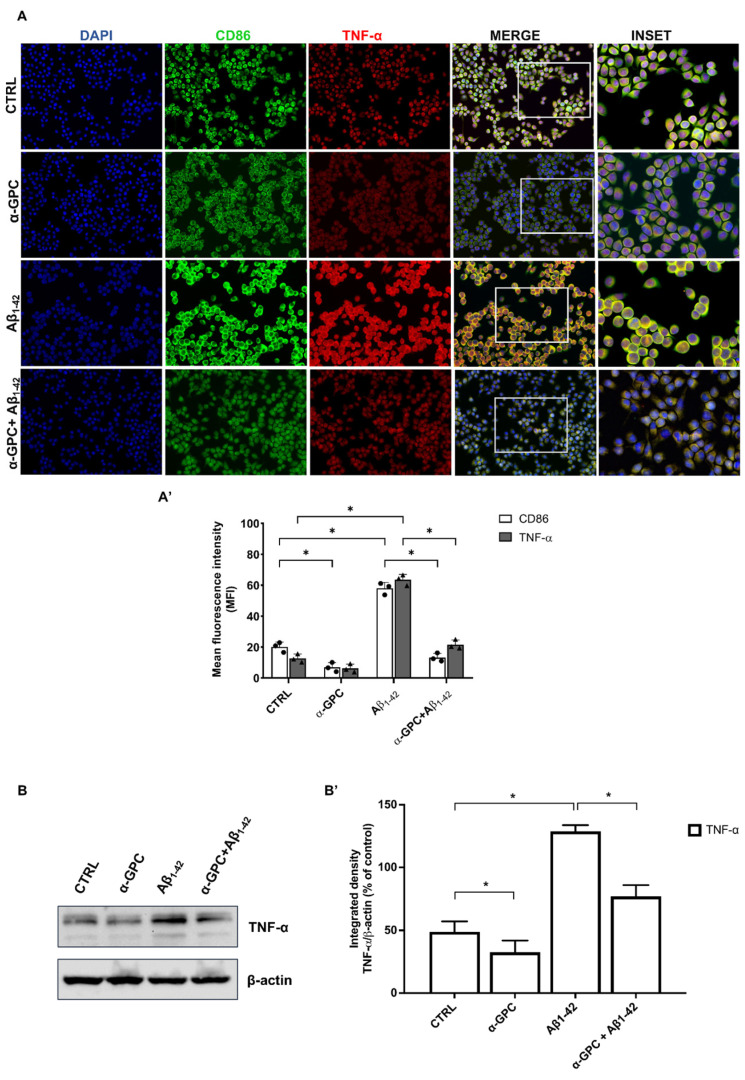
Pro-inflammatory microglia were blunted by α-GPC treatment. (**A**) Representative images (original magnification 20×; inset 40×) of the fluorescent immunocytochemical detection of CD86 and TNF-α expression in BV2 cells pre-treated for 1 h with α-GPC (1 μM) and treated for 48 h with Aβ_1–42_ (5 μM) and the respective mean fluorescence intensity (MFI) analysis (**A’**), the boxes in the fourth column represent the selected magnified area shown in the fifth column (inset). (**B**) Western blot for TNF-α protein expression in BV2 cells and the respective densitometric analysis (**B’**). The data are expressed as means ± S.E.M. One-way ANOVA and the Bonferroni post-hoc test were used to determine statistical significance. * *p* < 0.05.

As shown in Figure 2, Aβ treatment significantly increased the expression of CD86 and TNF-α in BV2 cells, indicative of the M1 pro-inflammatory phenotype. In contrast, α-GPC treatment prior to Aβ stimulation significantly counteracted the Aβ-induced increase of CD86 and TNF-α (Figure 2). 

**Figure 3:**
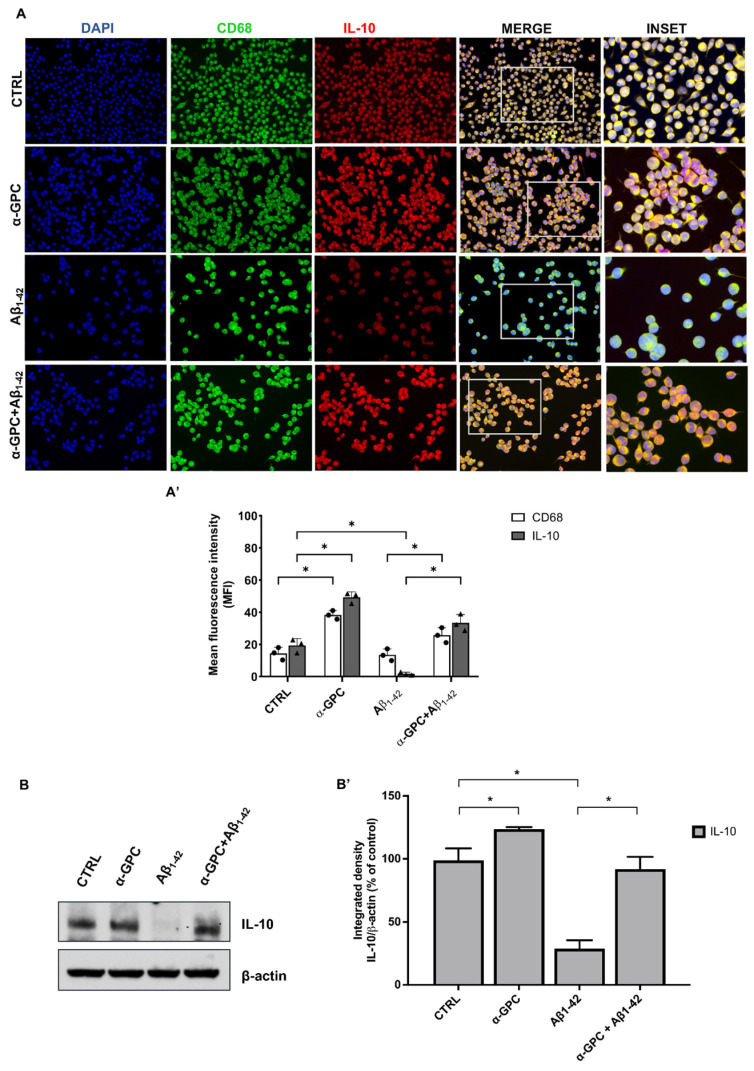
α-GPC contributes to the switching of microglia toward a pro-inflammatory phenotype. (**A**) Representative images (original magnification 20×; inset 40×) of the fluorescent immunocytochemical detection of CD68 and IL-10 expression in BV2 cells pre-treated for 1 h with α-GPC (1 μM) and treated for 48 h with Aβ_1–42_ (5 μM) and the respective mean fluorescence intensity (MFI) analysis (**A’**), the boxes in the fourth column represent the selected magnified area shown in the fifth column (inset). (**B**) Western blot for IL-10 protein expression in BV2 cells and the respective densitometric analysis (**B’**). The data are expressed as means ± S.E.M. One-way ANOVA and the Bonferroni post-hoc test were used to determine statistical significance. * *p* < 0.05.

Consistently, Aβ treatment decreased the expression of the anti-inflammatory markers CD68 and IL-10, whereas pre-treatment with α-GPC was able to increase their expression in the BV2 cells that underwent Aβ treatment (Figure 3). The Western blot analysis (Figure 2) further confirmed that the expression of TNF-α was substantially increased in BV2 cells challenged with Aβ_1–42_, whereas pre-treatment with α-GPC significantly attenuated its expression. On the other hand, the expression of IL-10 was reduced in BV2 cells challenged with Aβ_1–42_ and was significantly increased by pre-treatment with α-GPC (Figure 3).

In addition to the previously highlighted positive effects of α-GPC on BV2 cells subjected to Aβ treatment, it is noteworthy that α-GPC alone was able to regulate the protein expression of TNF-α and IL-10 compared to the control group (Figure 2 and Figure 3).

### 3.3. α-GPC Treatment Modulates α7 nAchR Expression in BV2 Cells

The expression of α7 nAChR in BV2 microglial cells is well-documented [19], and it is established that the activation of microglial α7 nAChR suppresses the production of several pro-inflammatory molecules [20]. To investigate the potential impact of α-GPC treatment on α7 nAchR expression in microglia exposed to Aβ, we performed fluorescent immunocytochemistry in BV2 cells challenged with Aβ_1–42_ (5 µM) for 48 h, either alone or after a 1 h pre-treatment with α-GPC (1 µM). 

**Figure 4:**
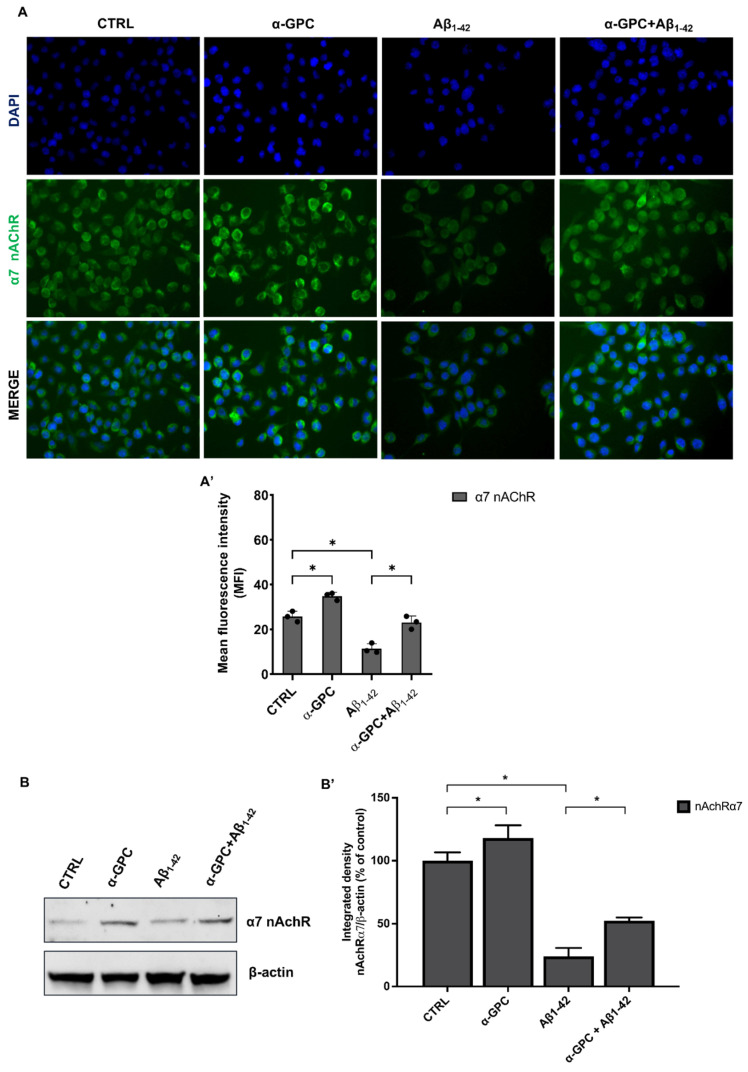
α7 nAchR is modulated by α-GPC treatment. (**A**) The fluorescent immunocytochemistry detection (original magnification 20×) of α7 nAChR in BV2 cells pre-treated with α-GPC (1 μM) and treated for 48 h with Aβ_1–42_ (5 μM) and the respective mean fluorescence intensity (MFI) analysis (**A’**). (**B**) Western blot for α7 nAChR protein expression in BV2 cells and the respective densitometric analysis (**B’**). The data are expressed as means ± S.E.M. One-way ANOVA and the Bonferroni post-hoc test were used to determine statistical significance. * *p* < 0.05.

Although BV2 cell cultures challenged with Aβ showed reduced expression of α7 nAChR compared to untreated cells, pre-treatment with α-GPC increased its expression (Figure 4). 

Consistent with these findings, the Western blot analysis of lysates of BV2 cells treated with α-GPC and/or Aβ_1–42_, conducted to verify the modulation of α7 nAChR expression, corroborated previous data (Figure 4). In addition to the positive effects of α-GPC on Aβ-treated BV2 cells, it is worth noting that α-GPC increased α7 nAChR protein expression compared to the control group.

### 3.4. Alpha-Bungarotoxin Prevents the Effect of α-GPC on α7-nAChR-Mediated [Ca^2+^]_i_ Increase and Inward Currents in Microglial Cells

To investigate whether the beneficial effects of α-GPC on microglia are mediated by the α7 nAChR, the BV2 cells were challenged with α-GPC or acetylcholine (Ach) alone and in combination with α-BTX, an antagonist of α7 nAChR, to measure the [Ca^2+^]_i_ and ACh currents.

**Figure 5:**
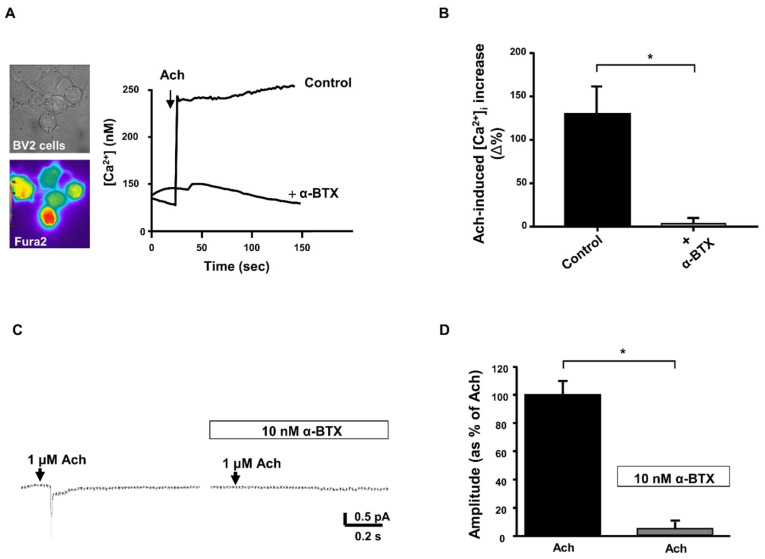
Effect of α-bungarotoxin on acetylcholine-induced [Ca^2+^]_i_ increase and acetylcholine-induced inward current in microglial BV2 cells. (**A**) Representative traces and quantification (**B**) for the effect of acetylcholine (Ach, 1 µM) alone or in the presence of α-bungarotoxin (10 nM) on [Ca^2+^]_i_, expressed as the Δ% increase peak over basal values (*N* = 40 cells for Ach and *N* = 35 cells for Ach + α-BTX). On the left, the representative brightfield and pseudocolor images of Fura-2 loaded BV2 cells are shown. (**C**) Representative current traces in response to Ach (1 µM) alone or in the presence of α-BTX (10 nM), and the quantification of current amplitude (**D**). The values are expressed as the mean ± SEM of three independent experimental sessions (*N* = 8 cells for Ach and *N* = 10 cells for Ach + α-BTX). * *p* < 0.05.

The results showed that Ach induced a significant increase in [Ca^2+^]_i_ in Fura2-loaded BV2 cells and a rapid inward current measured using patch-clamp electrophysiology, effects that were inhibited by treatment with α-BTX (Figure 5). 

**Figure 6:**
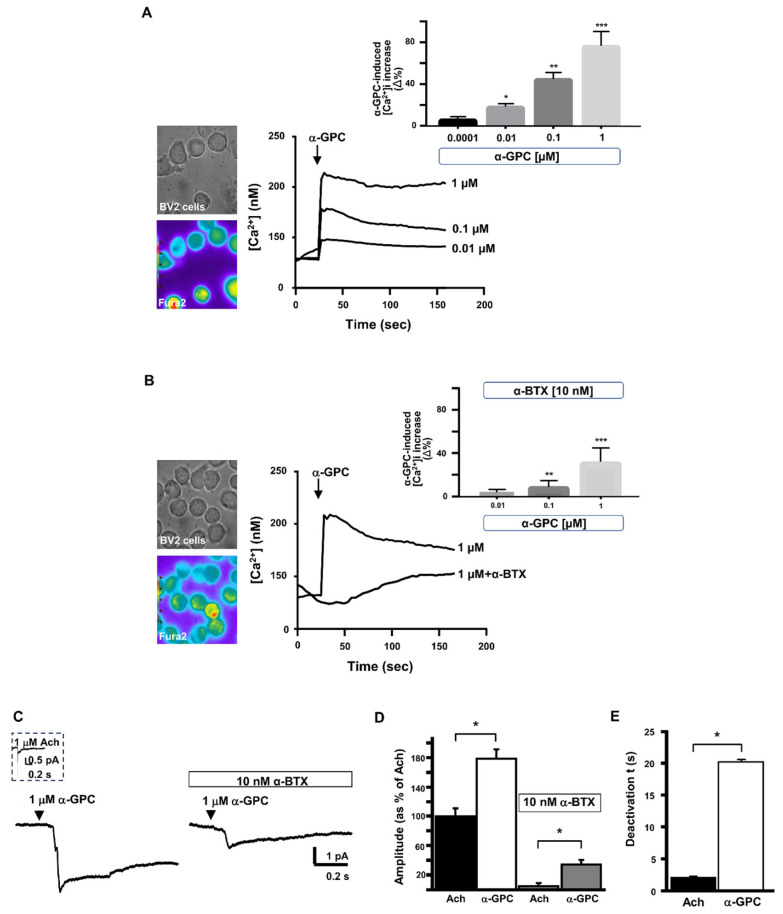
Effect of α-GPC on [Ca^2+^]_i_ increase and the α7-nAChR-encoded inward current in microglial BV2 cells. (**A**) Representative traces and quantification (**B**) for the effect of different concentrations of α-GPC (0.01–1 µM) alone or α-GPC (1 µM) + α-BTX (10 nM) on [Ca^2+^]_i_, expressed as the Δ% increase peak over basal values (*N* = 35 cells for α-GPC and *N* = 30 cells for α-GPC + α-BTX). On the left of each panel, the representative brightfield and pseudocolor images of Fura-2 loaded BV2 cells are shown. * *p* < 0.05 vs. control (basal values) and 0.0001 µM; ** *p* < 0.05 vs. control and 0.01 µM; *** *p* < 0.05 vs. all. (**C**) Representative traces of Ach-like currents elicited by α-GPC (1 µM) alone or + α-BTX (10 nM), and the quantification of current amplitude (**D**). The values are expressed as the mean ± SEM of three independent experimental sessions (*N* = 9 cells for α-GPC and *N* = 12 cells for α-GPC + α-BTX). (**E**) Quantification of the slow deactivation induced by α-GPC. * *p* < 0.05.

Interestingly, α-GPC was able to significantly increase the [Ca^2+^]_i_ in a concentration-dependent manner (Figure 6A), with the highest observed increase at the concentration of 1 µM. Moreover, α-BTX prevented the α-GPC-induced effect on [Ca^2+^]_i_ (Figure 6B), suggesting its ability to selectively modulate the α7 nAChR receptor in microglial cells. Indeed, α-GPC elicited an inward current that was found to be higher than that elicited by Ach, and this effect was blocked by α-BTX, as demonstrated by the patch-clamp-detected current amplitude (Figure 6C,D). Accordingly, the observed current was the result of delayed current inactivation (Figure 6E). 

### 3.5. Alpha-Bungarotoxin Reverts the Effects of α-GPC on Anti-Inflammatory Signaling Molecules

**Figure 7:**
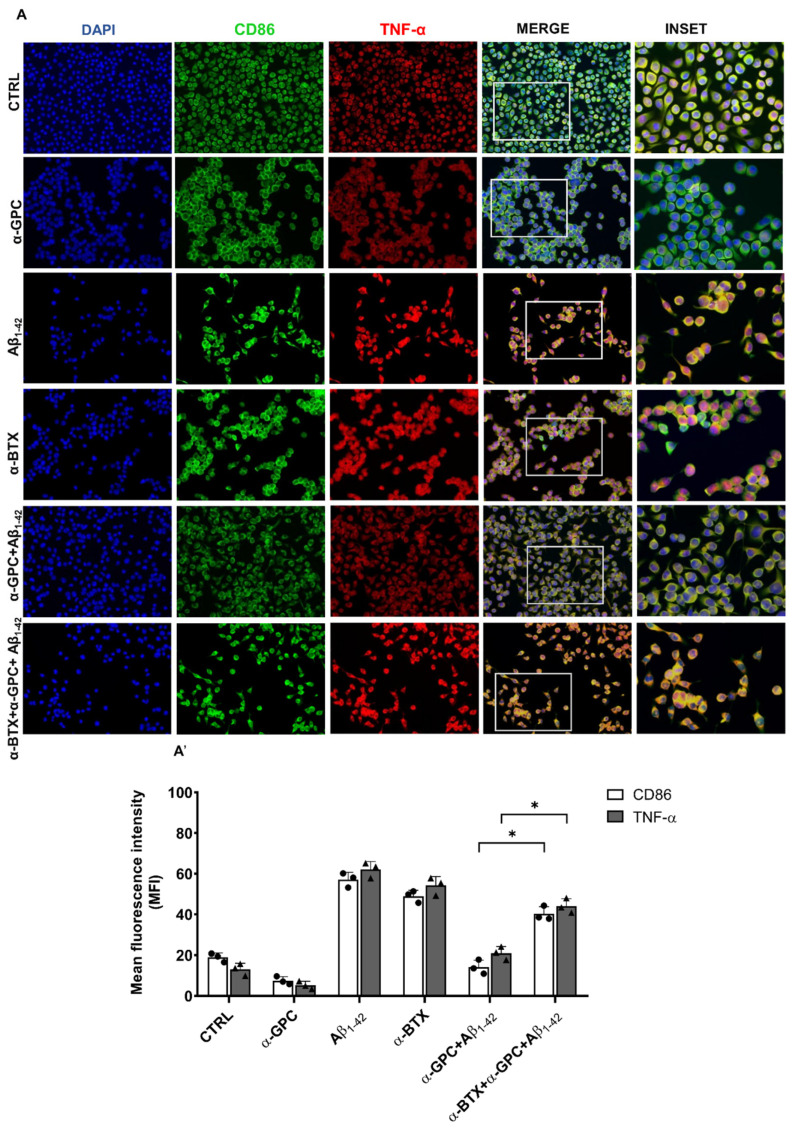
α-BTX restores the expression of pro-inflammatory molecules. (**A**) Representative images (original magnification 20×; inset 40×) of the fluorescent immunocytochemical detection of CD86 and TNF-α expression in BV2 cells pre-treated for 1 h with α-GPC (1 µM) and treated for 48 h with Aβ_1–42_ (5 μM)/α-BTX (100 nM) and the respective mean fluorescence intensity (MFI) analysis (**A’**), the boxes in the fourth column represent the selected magnified area shown in the fifth column (inset). The data are expressed as means ± S.E.M. Differences between the groups were considered significant at * *p* < 0.05 (One-way ANOVA followed by the Bonferroni post-hoc test).

To assess the role of α7 nAChR in the regulation of the anti-inflammatory effects mediated by α-GPC, we performed experiments on BV2 cells challenged with α-BTX, either alone or in the presence of α-GPC and/or Aβ_1–42_. The expression of pro-inflammatory markers CD86 and TNF-α, as well as the anti-inflammatory markers CD68 and IL-10, was evaluated using fluorescence immunocytochemistry. The results showed that α-BTX was able to inhibit the anti-inflammatory effect induced by α-GPC in BV2 microglial cells challenged with Aβ_1–42_, as demonstrated by the substantial increase in the expression of inflammatory markers (Figure 7).

**Figure 8:**
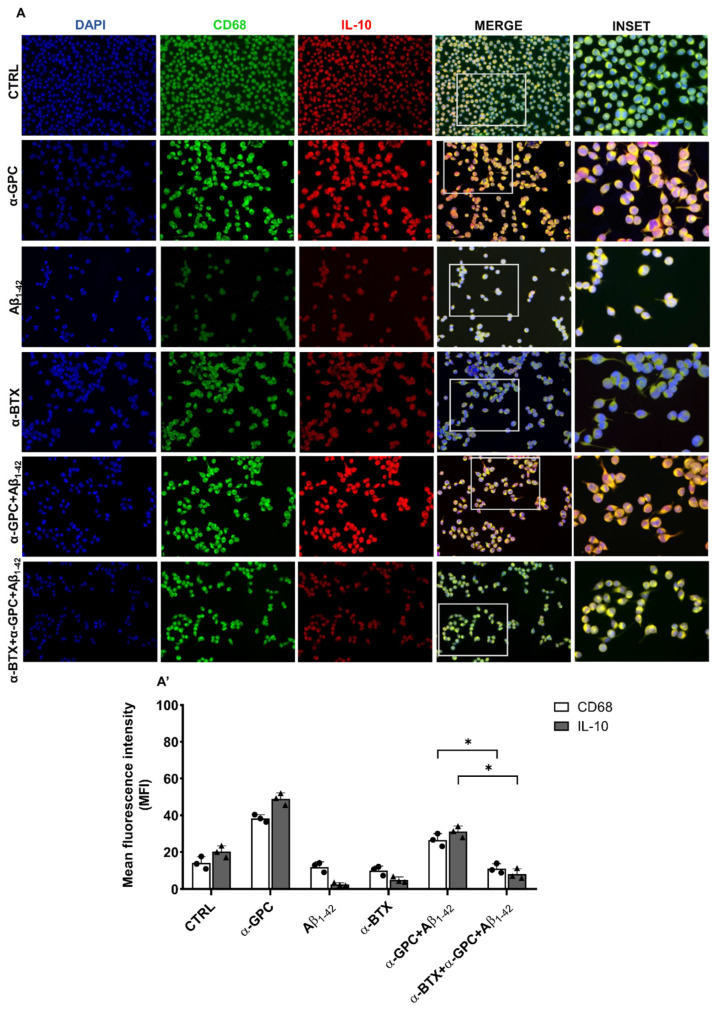
α-BTX inhibits the anti-inflammatory effects of α-GPC. (**A**) Representative images (original magnification 20×; inset 40×) of the fluorescent immunocytochemical detection of CD68 and IL-10 expression in BV2 cells pre-treated for 1 h with α-GPC (1 μM) and treated for 48 h with Aβ_1–42_ (5 μM)/α-BTX (100 nM) and the respective mean fluorescence intensity (MFI) analysis (**A’**), the boxes in the fourth column represent the selected magnified area shown in the fifth column (inset). The data are expressed as means ± S.E.M. Differences between the groups were considered significant at * *p* < 0.05 (One-way ANOVA followed by the Bonferroni post-hoc test).

On the other hand, α-BTX treatment blunted the expression of both IL-10 and CD68 in BV2 microglial cells pre-treated with α-GPC and challenged with Aβ_1–42_ (Figure 8).

## 4. Discussion

In the evolving landscape of AD therapeutics, characterized by treatments that primarily address symptoms with limited disease-modifying properties [21], our study explored the potential of choline alphoscerate (α-GPC).

Despite being available in the pharmaceutical market since 1987, the interest in α-GPC declined after the introduction of cholinesterase inhibitors. However, over the past decade, renewed attention has been given to this choline-containing phospholipid in several pre-clinical studies and clinical investigations [5,22,23]. 

In the present study, we investigated the effects of α-GPC, used as an indirect agonist of α7 nAchR, in an in vitro model of microglia in AD. The primary objective was to elucidate whether α-GPC holds dual protective capacity, not only safeguarding neuronal cells from Aβ-induced toxicity [24,25] but also orchestrating a concurrent protective effect in microglial cells challenged with Aβ_1–42_.

Our findings showcase that, although α-GPC did not interfere with cell survival, it effectively counteracted the detrimental effects induced by Aβ_1–42_ in BV2 microglial cells. 

Similar to Ach, α-GPC, which contains choline in its structure and may act as a precursor to Ach, can modulate microglial activity in response to Aβ, involving changes in cytokines release, phagocytic activity, and other microglial functions [26]. 

Our observations unveiled a remarkable aspect of α-GPC as it prompted a discernible transition in Aβ-activated microglia from the pro-inflammatory M1 phenotype to the protective M2 phenotype. Specifically, when BV2 microglial cells were exposed to Aβ_1–42_, they manifested a heightened pro-inflammatory state typical of the M1 phenotype, characterized by the high expression of CD86 and TNF-α. TNF-α, primarily produced by microglia, astrocytes, and neurons in response to various stimuli [27], represents a key molecule in orchestrating chronic inflammation. Indeed, TNF-α can have an impact on Aβ plaque synthesis and neurofibrillary tangle formation, fueling the progression of AD pathology [28,29]. 

Of note, α-GPC treatment in the presence of Aβ_1–42_ induced a distinct phenotypic shift towards the M2 status, characterized by a significant attenuation of the expression of inflammatory markers and a concurrent increase in the anti-inflammatory cytokine IL-10, a cytokine known to act on amyloid and reduce pro-inflammatory molecules [30].

Several studies indicate the presence of a parasympathetic mechanism that suppresses the release of inflammatory cytokines, thereby regulating inflammation. This mechanism, known as the “cholinergic anti-inflammatory pathway”, shows that acetylcholine inhibits the release of pro-inflammatory cytokines, such as TNF-α, IL-6, and IL-1β, in primary human macrophages [31]. Studies conducted using antisense oligonucleotides of the α7 subunit revealed α7 nAChR as the main player of the above-mentioned process [32], shedding light on its role in orchestrating the choline-mediated anti-inflammatory effects [33,34,35,36]. Indeed, it has been reported that α7 nAChRs expressed by glial cells may counterbalance the neuroinflammatory effects of Aβ fragments [37]. The activation of α7 nAChRs with a selective agonist has been reported to promote Aβ phagocytosis by cultured microglial cells [37] and improve cognitive function in an AD mouse model [38].

It is well established that among nicotinic receptors, the homomeric α7 subtype exhibits one of the highest Ca^2+^: Na^+^ permeability ratios [39]. Considering the multitude of cellular pathways regulated by calcium influx through this receptor, particularly those associated with neuroprotection [40,41], we measured [Ca^2+^]_i_. 

Delving into the specific role of α7 nAChR, known for its involvement in cognition [42], attention [43], and neuroprotection [44], we investigated the functional dynamics of α7 nAChR in BV2 microglia in response to α-GPC or acetylcholine (Ach), used as an endogenous agonist, either in the presence or absence of the α7-nAChR-specific inhibitor α-bungarotoxin (α-BTX). 

Interestingly, we observed that α-GPC, resembling the actions of Ach, induced a rapid inward current that was found to be higher than that elicited by the endogenous agonist Ach in BV2 microglia, and this dynamic effect was almost entirely blocked by the α7 inhibitor α-bungarotoxin (α-BTX). These observations further support the ability of α-GPC to selectively modulate the α7 nAChR receptors in microglial cells. 

In line with this, our results suggest that the choline-mediated anti-inflammatory effects are mediated by α7 nicotinic receptors and reversed by the selective α7 nicotinic receptor antagonist α-BTX [45].

## 5. Conclusions

In conclusion, our study suggests that α-GPC may play a significant role in counteracting inflammation associated with AD. This potential anti-inflammatory effect may be attributed to its ability to enhance cholinergic stimuli and activate the α7 nicotinic acetylcholine receptor system. Although further studies are needed to confirm its therapeutic use, α-GPC represents a promising strategy to mitigate neuroinflammation and AD pathology, considering its non-invasiveness and clinical tolerability [46]. 

## Data Availability

The data presented in this study are available from the corresponding author on reasonable request.
